# Gallstone Ileus With Cholecystoenteric Fistula in an Elderly Female: A Case Report

**DOI:** 10.7759/cureus.37077

**Published:** 2023-04-03

**Authors:** Jesús Pichardo, Joel Zapata, Radhanilda Echavarría, Raúl Ubiñas, Pedro Báez, Ángel Gómez

**Affiliations:** 1 Department of Surgery and Specialties, CEDIMAT (Centros de Diagnóstico y Medicina Avanzada y de Conferencias Médicas y Telemedicina), Santo Domingo, DOM; 2 Department of Gastroenterology, CEDIMAT (Centros de Diagnóstico y Medicina Avanzada y de Conferencias Médicas y Telemedicina), Santo Domingo, DOM

**Keywords:** cholecystoenteric fistula, small-bowel obstruction, pneumobilia, gallstone ileus, surgical case reports

## Abstract

Mechanical small-bowel obstruction can occur due to various reasons, including the impaction of a gallstone in the ileum after it has passed through a cholecystoenteric fistula. Gallstone ileus is an infrequent yet significant cause of this condition. This case report documents an instance of gallstone ileus, which accounts for less than 1% of patients with mechanical small bowel obstruction. We report a 75-year-old female patient who presented with colicky pain in both upper quadrants, hyporexia, and constipation that worsened during a period of nine days, which subsequently was accompanied by nausea and vomiting of bilious appearance in the next three days. Abdominal CT reported a dilated common bile duct (1.7 cm) with multiple stones inside measuring between 5 and 8 mm associated with pneumobilia of intrahepatic bile ducts and dilatation of small intestinal loops produced by a high-density image of approximately 2.5 cm. Laparoscopic exploration showed an obstructive mass measuring 15 cm from the ileocecal valve corresponding to a 2.54 x 2.35 cm gallstone, which was removed and enterorrhaphy was performed. The sine qua non condition for gallstone ileus to occur is the presence of a fistula between the gallbladder and the gastrointestinal tract. The treatment is mainly surgical and should be aimed primarily at the intestinal obstruction and secondarily at the cholecystoenteric fistula. This condition tends to have a high rate of complications and consequently long hospital stays. Making a timely diagnosis provides us with the tools for a surgical approach aimed at intestinal obstruction and subsequently in the management of the biliary fistula.

## Introduction

Mechanical small bowel obstruction can occur due to various reasons, including the impaction of a gallstone in the ileum after it has passed through a cholecystoenteric fistula. It is defined as a mechanical intestinal obstruction due to impaction within the gastrointestinal tract of one or more gallstones. Gallstone ileus occurs in fewer than 0.5% of patients who present with mechanical small bowel obstruction and it is most frequent in elderly patients and females [1].

Since this pathology is associated with relatively high rates of morbidity and mortality, a reflection of the advanced age of the patients, deteriorated clinical conditions, as well as the high incidence of concomitant diseases, an early and accurate diagnosis is very important [2].

In this report, we present a case of a 75-year-old female diagnosed with a bowel obstruction due to a gallstone that passed through a cholecystoenteric fistula. The patient was admitted and operated on in an academic hospital.

## Case presentation

A 75-year-old Latin female with a morbid history of arterial hypertension, arthritis, cholelithiasis, and obesity came to the emergency room due to colicky abdominal pain of 7/10 on a Numeric Rating Scale (NRS), with 0 being no pain and 10 being the worst pain imaginable. Additionally, she reported hyporexia and constipation that worsened during a period of nine days, which subsequently was accompanied by nausea and vomiting of bilious appearance in the next three days every time she ingested any food. During the initial assessment, the patient reported urinating only once all day, suggesting a decreased urine output and indicating that she was experiencing oliguria. The patient reported being compliant with her medications, which included atenolol, chlorthalidone, aspirin, and celecoxib. The patient denied any recent changes to her medications or diet. On physical examination, the patient looked critically ill, with a depressible abdomen, tenderness on superficial and deep palpation in all the abdomen diffusely, a negative Murphy's sign, no peritoneal irritation, and no masses or visceromegalies. The patient's vital signs were recorded as follows: blood pressure of 130/80 mmHg, heart rate of 78 beats per minute, respiratory rate of 16 breaths per minute, and body temperature of 37.1°C. Oxygen saturation was measured at 100%.

The initial laboratory test results were obtained and are presented in Table 1.

**Table 1:** Laboratories test results Abnormal values are shown in bold. AST: aspartate aminotransferase; ALT: alanine aminotransferase; SGOT: serum glutamic oxaloacetic transaminase; SGPT: serum glutamic pyruvic transaminase.

Test	Case result	Normal range
White blood cells (WBCs)	11.5 x 10³U/L	4,000-11,000 cells/μL
Neutrophils	64.3%	40-60% of the WBC count
Lymphocytes	20.7%	20-40% of the WBC count
Eosinophils	0.3%	1-6% of the WBC count
Monocytes	14.4%	2-8% of the WBC count
Basophils	0.3%	0.5-1% of the WBC count
Hemoglobin	12.0 g/dL	12-16 g/dL
Hematocrit	34.1%	36-48%
Platelets	313 x 10³/uL	150,000-450,000 cells/μL
Serum creatinine	3.64 mg/dL	0.6-1.3 mg/dL
Blood urea nitrogen (BUN)	68.1 mg/dl	7-20 mg/dL
Sodium	137 mmol/L	135-145 mmol/L
Potassium	4.8 mmol/L	3.5-5.0 mmol/L
AST (SGOT)	73 U/L	5-40 U/L
ALT (SGPT)	75 U/L	7-56 U/L
Total bilirubin	0.52	0.1-1.2 mg/dL
Direct bilirubin	0.33 mg/dl	0-0.3 mg/dL
Amylase	100 U/L	30-110 U/L
Lipase	378 U/L	0-160 U/L
Prothrombin time (PT)	14.9 seconds	11-13.5 seconds
International normalized ratio (INR)	1.09	0.8-1.2 (INR)
Activated partial thromboplastin time (aPTT)	28.2 seconds	25-35 seconds

**Figure 1:**
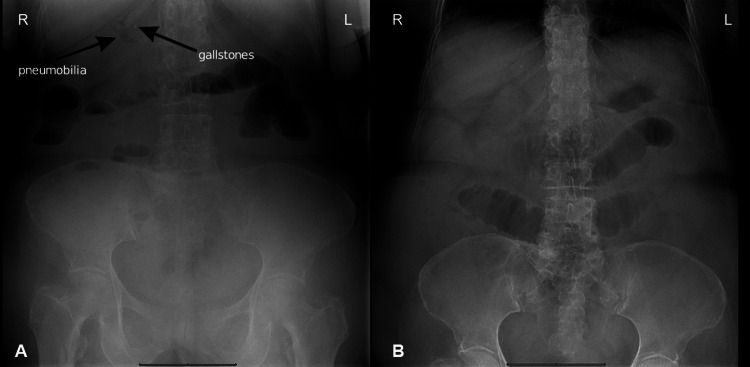
Plain abdominal radiograph: (A) standing and (B) sitting

An abdominal X-ray was done showing dilation of intestinal loops associated with air-fluid levels, absence of distal intracolonic air, and well-defined radiopacity projected in the right hypochondrium (Figure 1).

**Figure 2:**
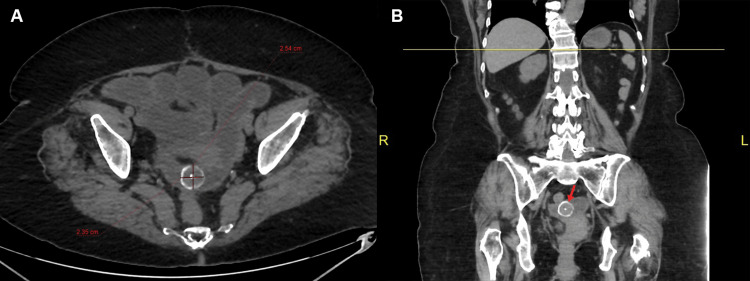
Non-contrast-enhanced computed tomography showing a 2.35 x 2.54 cm object inside intestinal loops (red arrow)

**Figure 3:**
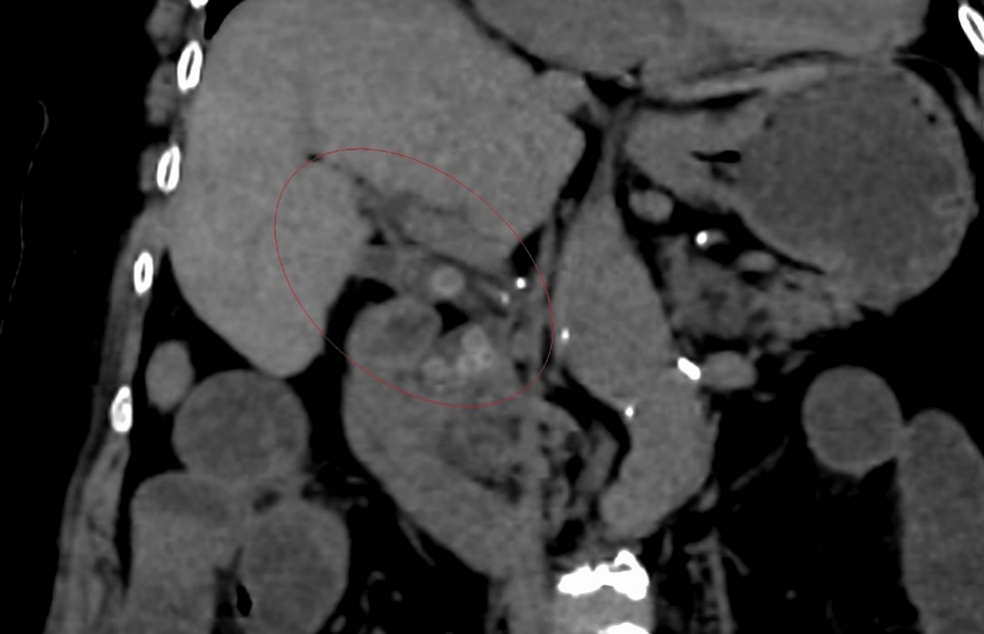
Non-contrast-enhanced computed tomography showing a cholecystoenteric fistula and multiple gallstones (red circle)

Abdominal CT reported a dilated common bile duct (1.7 cm) with multiple stones inside that measure between 5 and 8 mm associated with pneumobilia of intrahepatic bile ducts. Dilatation of small intestinal loops produced by a high-density image of approximately 2.5 cm, identified in the hypogastrium topography. Also, few diverticula in the ascending and sigmoid colon and free fluid in the pelvic cavity were noted (Figures 2, 3).

**Figure 4:**
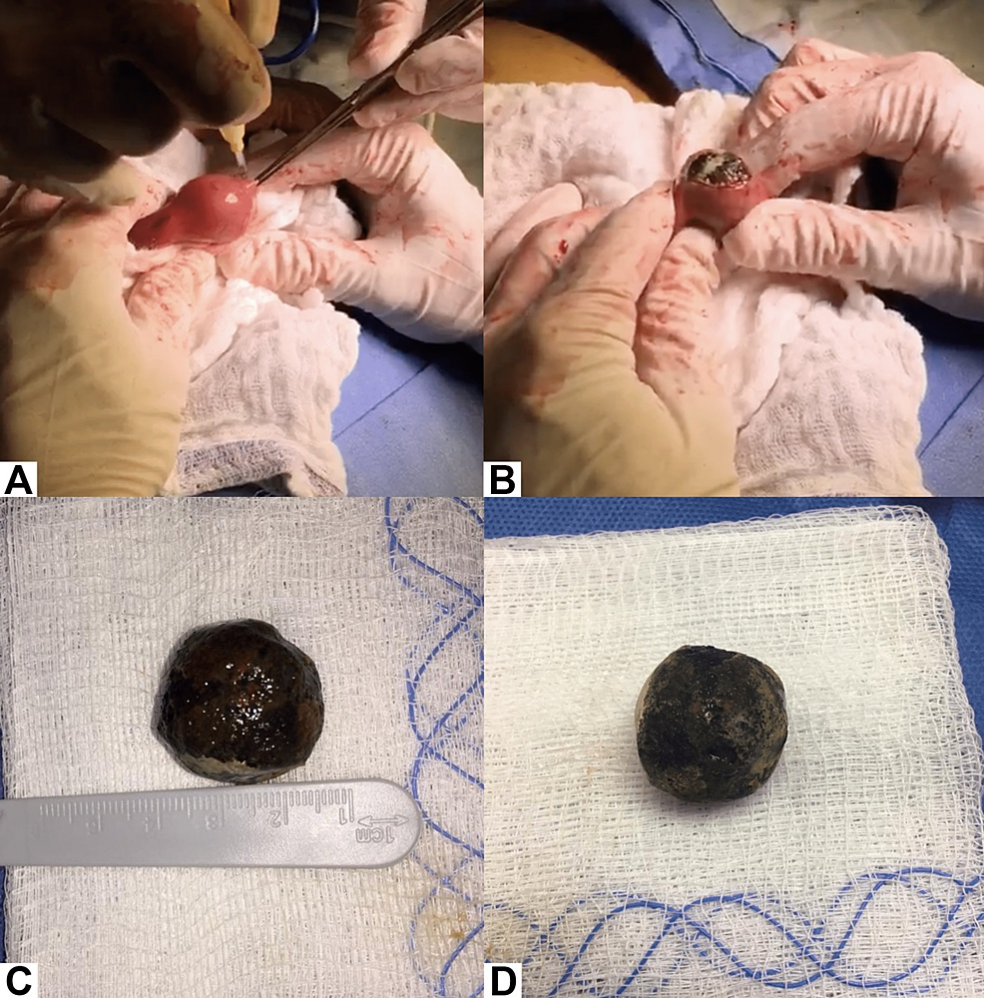
Removal of a 2.54 cm gallstone obstructing ileum (A) Longitudinal incision of the ileum at the site of obstruction. (B) Extraction of the gallstone. (C & D) Gallstone.

The patient was admitted by the general surgery department with a diagnosis of intestinal obstruction and surgical treatment was decided. The patient was properly hydrated with 0.9% saline via IV. The surgical approach was by exploratory laparoscopy, finding an obstructive mass 15 cm from the ileocecal valve corresponding to a 2.54 x 2.35 cm gallstone. The surgical technique used in this case involved an initial incision made on the right iliac fossa (RIF), followed by an exploration of the intestinal loops until the obstructive mass was located. Once the mass was identified, a longitudinal incision was made on the segment of the obstruction. The gallstone causing the obstruction was then carefully removed, and the segment was repaired using a technique called enterorrhaphy. This involved suturing the incision made on the bowel segment, thereby restoring the continuity of the intestinal lumen (Figure 4). The primary aim was to resolve the obstruction initially while deferring the management of the fistula to a subsequent surgical intervention, which would entail a cholecystectomy. The patient came out of surgery with a bladder catheter, a nasogastric tube, and a Jackson-Pratt drainage with serohematic content.

**Figure 5:**
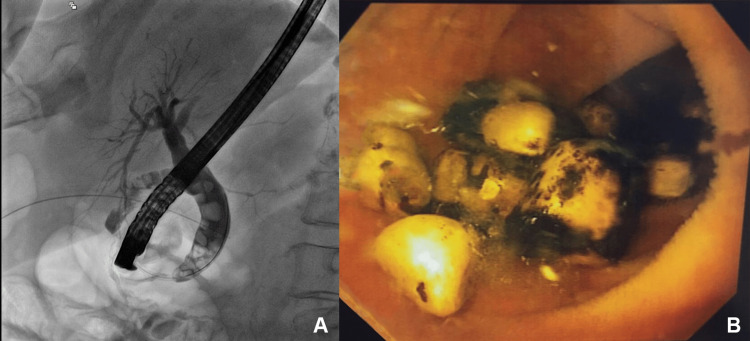
Endoscopic retrograde cholangiopancreatography showing multiple gallstones (A) Abdominal X-ray showing multiple gallstones from the common bile duct to the gallbladder. (B) Multiple gallstones inside the intestinal loop after dilation of the bile duct.

An endoscopic retrograde cholangiopancreatography (ERCP) was performed on the third day after surgery, where choledocholithiasis and an image suggestive of a gallbladder-dependent bilioenteric fistulous tract were observed, and approximately 11 stones were extracted (Figure 5). On the fourth day after surgery, stools were evident on several occasions with peristalsis present, so a liquid diet was started. On the seventh postoperative day, drainage was removed. After tolerating a soft solid diet, the patient was discharged on the eighth postoperative day with a follow-up appointment in seven days.

Seven months later, the patient underwent cholecystectomy by laparotomy with the closure of the cholecystoenteric fistula without any complications. The surgical technique involved making a right subcostal incision to access the abdominal cavity. The surgeon carefully evaluated the extent of the fistula and located the site of the cholecystoenteric fistula. The adjacent tissues were then meticulously dissected to expose the opening. The borders of the fistula were trimmed, and the opening was sutured shut with care. The surgeon then proceeded to locate the gallbladder, identified the cystic duct and artery, and dissected them meticulously. The gallbladder was detached from the liver bed with caution to avoid any damage to surrounding structures. The site was checked for any signs of bleeding or leakage before closing the right subcostal incision with sutures. The patient was kept under observation in the recovery room before being transferred to a hospital room for postoperative monitoring. No complications were reported.

## Discussion

Gallstone ileus is a rare complication of biliary pathology and occurs in 0.3% to 4% of patients with cholelithiasis [3]. According to recent studies, it has been found that the incidence of small bowel obstruction due to this condition is relatively low in patients under 65 years of age, with less than 4% of cases being reported. However, as patients age, their risk of developing this condition increases significantly, with a significant rise in incidence reported in patients aged 65 years or older, where the incidence rate is approximately 25% [4,5]. Its prevalence is higher in women, with a female-to-male ratio of 3.5-3.6:1 [3]. Due to the advanced age of the patients, coexisting medical conditions, delayed hospital admission, and postponed therapeutic intervention, the morbidity rate associated with gallstone ileus can reach 50%, while the mortality rate ranges from 12 to 27%. [3-6].

The sine qua non condition for gallstone ileus to occur is the presence of a fistula between the gallbladder and the gastrointestinal tract [1]. The pathogenesis involves a concurrent episode of acute cholecystitis. The inflammation in the gallbladder leads to adhesion formation between the surrounding structures [1]. The pressure effect of the gallstone leads to necrosis and erosion through the wall of the gallbladder and the fistula formation, with cholecystoduodenal fistula being the most frequent type; however, cholecystocolonic and cholecystogastric fistulas can also result in gallstone ileus. When the gallbladder is free of calculi, it becomes a blind sinus tract and contracts down to a small fibrous remnant [1].

In a 32-year retrospective review of 24 cases, 90% of obstructing stones were greater than 2 cm in diameter, with the majority measuring over 2.5 cm [7]. The minimum diameter of the stone necessary to produce intestinal obstruction is 2.5 cm unless there is an alteration of the previous intestinal dynamics or some cause of stenosis [8]. In classic gallstone ileus, the stone is more frequently impacted in the distal ileum (70%), followed by the proximal ileum and jejunum (25%), colon in less than 4.8%, and duodenum in 5% (Bouveret’s syndrome) [9]. In the small bowel, the gallstone usually goes to the most distal parts and may become impacted and cause an obstruction, which are the terminal ileum and the ileocecal valve because of their relatively narrow lumen and less active peristalsis. The factors that determine the impaction are the size of the gallstone, the site of fistula formation, and the bowel lumen [10].

Clinical symptoms vary, depending on the site of the obstruction. The most common presenting clinical picture is a mechanical intestinal obstruction with abdominal distention and pain, vomiting, constipation or obstipation, and fluid imbalance. The patient may also have jaundice. In cases of chronic evolution, there will be recurrent episodes of pain caused by the passage of gallstones through the intestine, along with a period of asymptomatic time, reaching complete obstruction in several stages. During the abdominal exploration, signs include distension and increased bowel sounds. Physical examination and laboratory tests do not point to a particular cause of intestinal obstruction. Laboratory studies may show an elevated white blood cell count, which was the case in this patient with a slightly elevated count of 11.5 x 10³U/L, an abnormal liver function test, which was also the case, with elevated levels of aspartate aminotransferase (AST) and alanine aminotransferase (ALT) of 73 U/L and 75 U/L, respectively [3]. Additionally, it has been reported that an electrolyte imbalance may be present in very few cases [3]. However, in this case, such an imbalance was not found, which further supports the negative diagnostic significance of this finding.

Plain abdominal radiography frequently demonstrates a nonspecific pattern of intestinal obstruction and its diagnostic utility in the identification of gallstones is limited. Ultrasonography is most helpful in demonstrating the impacted stone as well as in confirming residual cholelithiasis or choledocholithiasis. A CT scan can objectify intestinal obstruction by identifying the stone and the level of obstruction, having a sensitivity greater than 90% [11]. Rigler's triad is the set of diagnostic imaging features used to diagnose gallstone ileus, which involves the distention of intestinal loops, the presence of radiopaque stones (in fewer than 10% of cases), and pneumobilia (known as Gotta-Mentschler sign) [3]. The diagnosis can be established with the presence of two out of these three signs [3].

The treatment is mainly surgical and should be aimed primarily at the intestinal obstruction and secondarily at the cholecystoenteric fistula and may be performed simultaneously or not depending on the patient's condition [3,11]. Intestinal obstruction is addressed with an enterolithotomy via laparoscopy or laparotomy. Cholelithiasis and cholecystoenteric fistula are generally treated in a second surgical stage (or concomitantly with enterolithotomy in low-risk patients), with a combined biliary procedure, which involves cholecystectomy and closure of the fistula [12]. Compared with enterolithotomy alone, the one-stage procedure reduces recurrences of gallstone ileus; prevents malabsorption and weight loss from a persistent cholecystoenteric fistula; and prevents cholecystitis, cholangitis, and gallbladder carcinoma, but with the risk of greater surgical morbidity and mortality [13].

## Conclusions

Gallstone ileus is a rare entity that can cause intestinal obstruction mainly in elderly women, with a high rate of complications and consequently long hospital stays. This case report highlights the importance of considering gallstone ileus as a potential diagnosis in patients with a history of cholecystitis and symptoms of bowel obstruction. It also emphasizes the value of utilizing imaging studies to confirm the diagnosis and identify any potential underlying causes. Furthermore, our report underscores the need for timely surgical intervention to prevent complications and improve patient outcomes. Making a timely diagnosis provides us with the tools for a surgical approach aimed at intestinal obstruction and subsequently in the management of the biliary fistula.
